# Supplementary material for: Are carbon emissions trading and green financial instruments synergistic? -Comprehensive quantitative research based on content analysis
Source: PLoS One. 2024 Mar 7;19(3):e0298601. doi: 10.1371/journal.pone.0298601 (PMC10919601; doi:10.1371/journal.pone.0298601)
Supplement: S1 Data — (ZIP) [file pone.0298601.s001.zip › Data/地级市Z_F工作报告.zip!/╔╜╢1⁄2/═■║ú╩╨/└┤╫╘╣1⁄2╓┌║┼í╛┬φ┐╦╩2╛▌═°í┐_2017.docx]

各位代表：

　　现在，我代表威海市人民政府向大会报告工作，请予审议，并请市政协各位委员提出意见。

一、政府工作回顾

　　2016年，在以习近平同志为核心的党中央坚强领导下，市政府全面贯彻党中央、国务院和省委、省政府决策部署，认真落实市委各项工作要求，紧紧依靠全市人民，牢固树立和贯彻落实新发展理念，坚持稳中求进工作总基调，统筹做好稳增长、促改革、调结构、惠民生、防风险各项工作，经济社会保持平稳健康发展。全市生产总值3212.2亿元，增长8%；一般公共预算收入260.5亿元，增长10.2%；城镇居民人均可支配收入39363元，农村居民人均可支配收入17573元，分别增长8.3%和7.7%，主要经济指标增幅保持全省前列。

　　一是深入实施创新驱动发展战略，经济发展新动能加速形成。坚持“产业强市、工业带动、突破发展服务业”，出台支持科技创新、品牌建设、生产性服务业发展等意见，设立政府引导基金，规划建设五大重点创新产业园，助推企业创新发展、产业转型升级。引进建设工信部电子信息技术综合研究中心、山东省碳纤维技术创新中心等重大创新平台。成为全省首批军民融合深度发展示范市。威高集团获得首届世界工业设计大会金奖、第四届中国工业大奖，威达机械成为全国首批制造业单项冠军示范企业，三角轮胎获得中国标准创新贡献奖。新增山东名牌产品17个、山东省服务名牌14个。高新技术产业产值比重达到40%，提高1.1个百分点。创建国家全域旅游示范区，旅游消费增长14.1%，信息消费增长20%以上，电子商务交易额增长39.8%，社会消费品零售总额增长11.1%。远洋渔业产量增长31.2%，休闲渔业产值增长33%，现代果业规模居全省第一，被整建制认定为国家农产品质量安全市。

　　二是扎实推进供给侧结构性改革，“三去一降一补”任务有效落实。积极稳妥化解过剩产能，轮胎、玻璃行业产能利用率分别达到81.3%和81.1%；推动造船企业重整重组，重点船舶企业造船完工量增长23%。商品房交易面积增长15.5%，房地产库存去化周期逐步缩减。有效处置企业资金链、担保圈风险，金融机构不良贷款余额、比率实现双降。全面落实减税降费各项政策，尽最大努力降低企业成本、减轻企业负担。

　　三是深入推进重点领域改革，实体经济发展支撑力切实增强。综合运用小微企业贷款风险补偿基金等政策，引导金融机构加大对实体经济支持力度。支持企业扩大直接融资，495家规模企业完成规范化公司制改制，三角轮胎、家家悦集团成功上市，新增新三板挂牌企业9家、区域性股权交易市场挂牌企业49家；全市直接融资213亿元，增长80.8%，占社会融资总量的53.2%，规模和结构创历年最好水平。全省首家民营银行蓝海银行获批筹建。农村信用合作社银行化改革全面完成。全面实施不动产统一登记制度。建成全市统一的公共资源交易平台。设立山东海洋产权交易市场威海分中心。获批创建全国社会信用体系建设示范城市，公共信用信息平台投入运营。深化城市公立医院改革，成为国家中医药综合改革试验区，获批全国健康城市试点、国家级医养结合试点、居家和社区养老服务改革试点。

　　四是积极开展先行先试，对外开放进一步扩大。开放试点工作再获新突破，被确定为国家服务贸易创新发展试点，威海综合保税区获批设立。7个港口、34个泊位获批对外开放。国家出口日韩食品“同线同标同质”交易公共服务平台在全国第一家线上线下同步运营。服务贸易支行挂牌运行。新引进116个服务业项目，世界500强企业软银集团服务外包基地落地实施。入选山东省跨境电子商务综合试验区，跨境电商出口货值7304万美元，是上年的4.1倍，规模居全省首位。全市外贸进出口总额1190.5亿元，增长13%。实际到账外资79.9亿元，增长15%。

　　五是扎实推进重点区域开发建设，全域城市化、市域一体化进程加快。东部滨海新城开发建设全面提速，“四横三纵”主干路网逐步完善，市委党校新校、市档案中心、市职业中等专业学校、公交新城中心站等重点工程主体完工，五渚河生态城、新城棚户区改造工程加快推进，国际医疗健康产业城、中韩自贸区经贸交流中心等产业项目启动建设。成功入选国家地下综合管廊建设试点城市。石岛湾核电站、文登抽水蓄能电站、龙山湖、八河水库增容工程等重点项目建设顺利推进，香水河大桥建成通车。全市固定资产投资增长13.2%，其中基础设施建设投资增长22.6%。加快推动“三个市民化”，户籍人口城镇化率提高到57.3%，常住人口城镇化率提高到65%。

　　六是全面落实绿色发展理念，生态环境质量持续优化。开展空气质量全面优化行动，省测“蓝天白云、繁星闪烁”天数359天，空气质量优良率89.9%，提高2个百分点，保持全省第一。PM2.5、PM10平均浓度达到国家二级标准；二氧化硫、二氧化氮平均浓度达到国家一级标准，在全省率先实现环境空气质量全面达到国家二级标准。加强海岸带综合整治，被确定为国家蓝色海湾整治行动城市。整治集中式饮用水水源保护区畜禽养殖场371处，治理中小河道及城郊河道95.7公里。全面完成农村环境综合整治，城乡环卫一体化群众满意度保持全省第一。

　　七是着力保障和改善民生，人民群众获得感稳步提升。全市所有建档立卡贫困人口提前实现省定贫困线脱贫，180个省定扶贫工作重点村中有120个达到退出标准。各类社会保险和救助扩面提标，居民基础养老金标准、医保补助标准、城乡低保标准、农村五保对象供养标准都有新提高。实施28个老旧住宅小区综合整治，完成棚户区改造2万户。各级政府累计投资28.1亿元解决城镇中小学“大班额”问题，新建改扩建学校38所，新增学位3.1万个。新建改扩建公办幼儿园15所，新增学位4160个。山东交通学院威海校区建成启用。实施智慧城市建设示范项目，市民卡、市民网受益群众不断增多，肉菜流通追溯体系投入运行，公共服务一体化信息便民工程建设经验在全国推广。

　　八是协调发展各项社会事业，社会保持和谐稳定。文明城市建设成果不断巩固提升，全国文明城市年度测评复查成绩全省第一。规划建设19个文化产业公共服务平台，公共文化服务数字化平台建成启用。推行医保卡支付健身消费，建成31处镇级全民健身中心。成功举办长距离铁人三项世界系列赛、威海—仁川帆船拉力赛等赛事。起草4部实体性地方性法规，其中2部经市人大常委会颁布实施，出台2部政府规章。加强普法宣传，被评为全国法治宣传教育先进城市。累计投入7.9亿元配强应急救援装备，投入3.8亿元健全城市公共安全视频监控网络，安全生产、食品药品安全、社会治安等形势总体平稳。国防动员、人民防空、民兵预备役工作进一步加强，被评为全国人民防空先进城市。民族、宗教、妇女儿童、外事、侨务、对台、档案、史志、地震、气象、仲裁、慈善和对口支援、扶贫协作等工作取得新进步。

　　各位代表，本届政府履职五年来，在市委的坚强领导下，坚持依法行政、高效施政、从严治政，大力推进法治政府、服务型政府、廉洁政府建设，主动适应把握引领经济发展新常态，着力推动经济转型升级，提高公共服务能力和水平，聚焦发展所需、群众所盼、短板所在，以改革破除发展瓶颈、以创新释放发展动力、以实干厚植发展优势，开创了现代化幸福威海建设新局面。

　　——在落实新发展理念上展现新作为。坚持生态立市、科教兴市、产业强市，千方百计稳定经济增长、推动转型升级、提高发展质量效益，全市生产总值年均增长9.3%，一般公共预算收入年均增长15.4%。三次产业比重由8.1：54：37.9调整为7.1:45.6：47.3。传统产业加快转型，战略性新兴产业占比逐年提高。现代服务业突破发展，新型业态不断涌现。高效生态特色农业快速发展，成为国家现代农业示范区。生态、生产、生活“三生共融”理念有效落实，生态环境比较优势日益突出，荣获环保领域最高社会奖——中华环境奖，科学发展综合考核保持全省前列。

　　——在市域一体发展上展开新布局。坚持全域城市化、市域一体化，全面启动重点区域开发建设，实施东部滨海新城、文登同城化、南海新区、乳山产业结构四个“三年突破”战略，文登撤市设区，临港区升级为国家级经济技术开发区，高区列入山东半岛国家自主创新示范区，经区成为国家产城融合示范区，荣成海洋生物产业园获批省级高新区和国家农业科技园区，环翠区北海旅游度假区获批省级旅游度假区，国家新型城镇化综合改革试点工作扎实推进，各城市组团协同联动发展，拉开了全域统筹的城市发展大框架，走出了威海特色的城市发展道路。

　　——在助推创新创业上取得新成果。坚持把创新作为引领发展的第一动力，全面优化创新环境、激发市场活力，科技创新投入持续加大，财政科技投入增长2.4倍，全社会研发经费增长2.1倍；国家海产品质检中心、山东船舶技术研究院等重大创新平台投入使用，省级以上科技创新平台数量增长69.8%，省级以上科技企业孵化器、创业孵化器数量增长6.3倍；率先建成创业大学，培训创业人员3.5万人；引进北京交通大学等知名高校，建成威海海洋职业学院，与170家高校科研院所建立产学研合作关系；蓝色产业领军人才团队、泰山系列人才工程专家数量实现突破性增长，新增各类人才14.8万人；高新技术企业数量增长1.5倍，科技型中小企业数量增长2.3倍，新登记企业数量增长2.5倍，市场主体总量翻了一番，全社会的创造活力竞相迸发，大众创业、万众创新成为经济增长的新引擎、转型升级的新动能。

　　——在深化改革开放上增创新优势。坚持以改革创新的思路和方法破解难题、推动发展，主动承担国家、省级改革试点53项，累计完成改革事项590项；组建城投、水投、交投、国有资本运营和新城股份公司，构建了市场化投融资机制；农村三项改革基本完成，农业农村发展活力进一步增强；基础教育综合改革、公立医院综合改革、文化体制改革都走在前列，各项社会事业发展机制不断创新；特别是在对外开放方面，我市被确定为中韩自贸区地方经济合作示范区、中欧城镇化合作示范城市，贸易便利化、功能性口岸建设、跨境电商等领域一系列先行先试政策落地实施，彰显了改革开放先行区的地位和优势。

　　——在补齐基础设施短板上实现新跨越。实施一大批事关长远发展的重大项目，青荣城际铁路全线贯通，威海正式进入“城铁时代”；威海机场完成升级改造，旅客吞吐量由93.5万人次增加到173万人次，迈入中型国际机场行列；威海港国际客运中心完成搬迁，对外开放港口数量增长1.8倍；石烟线外移工程、荣文高速公路建成通车，实现“县县通高速”；新建改造农村公路2600公里，实施农村公路安全生命防护工程635公里；市区两大综合交通枢纽投入使用，实现多种交通方式“零换乘”；公交智能化、环保化改造全面完成，城乡公交一体化实现全覆盖；胶东调水工程建成并正式通水，形成本地水与客水“双保障”的供水格局；入选首批国家智慧城市试点、信息惠民国家试点城市、宽带中国示范城市，信息化社会指数位居全国城市前列；各类基础设施全面提档升级，有力地破解了瓶颈制约，夯实了发展基础。

　　——在增进民生福祉上取得新突破。坚持“为了群众要真心、依靠群众要真诚、服务群众要真干”，从群众身边的点滴小事抓起，倾听群众呼声，办好民生实事，养老保险、医疗保险在全国率先实现城乡统筹、市域统筹，各项社会保障和救助标准全省领先，城镇登记失业率保持全省最低；公立医院全部取消药品加成，基层医疗机构全部实施基本药物制度，7家医院晋升三级甲等医院，实现从无到有新突破；构建“租售补”相结合的住房保障体系，帮助1.3万户住房困难家庭圆了“安居梦”；建设农村新型社区120个，受益居民3.4万户；中小学全部实现办学条件标准化，农村中小学新模式校车实现全覆盖，普惠性幼儿园比例由58%提高到80%，学校体卫艺教育全面加强，在全国率先实现义务教育均衡发展；城乡社区全部配备体育健身设施、公共文化设施，打造15分钟公共健身圈、文化休闲圈；财政民生支出比重提高到83.9%，教育、医疗卫生、文化投入分别增长2.2倍、2倍和2.7倍，公共服务均等化水平走在全省前列。

　　——在促进社会文明和谐上迈上新台阶。坚持文化引领、文明共建，持续开展文明创建活动，推进“君子之风·美德威海”建设，被评为首批文化强省建设先进市、全省“四德”工程建设示范市；海陆一体平安建设经验在全国推广，“双安双创”威海模式成为全国样板；第七次荣获全国双拥模范城；社会治安群众满意度保持全省首位；全市人民齐心协力、共建共享，以地级市第一名成绩成功创建全国文明城市。

　　——在建设人民满意政府上取得新成效。坚持简政放权、放管结合、优化服务，按照“让企业和群众不托人也能好办事、快办事、办成事”的工作要求，加快推进政府职能由“管理型”向“服务型”转变。推行模块化审批、联审联批等行政服务流程再造，市级行政审批事项压减52.9%，行政事业性收费事项压减69.6%，行政审批服务时限压缩51.8%，以政府权力的“减法”换取市场活力的“乘法”。大力推进“互联网+政务服务”，整合公共服务热线，建立政务服务、应急联动、便民服务三个热线平台，全面推行行政机关首问负责制，建立健全群众诉求转办督办机制，第一时间归口办理、回复落实，群众来信来电办结率保持在99.7%以上。健全行政决策公众参与、风险评估和合法性审查制度，全面加强政务公开，政府部门开通政务微信、微博，畅通社情民意表达渠道。主动接受人大的法律监督、政协的民主监督、媒体的舆论监督、群众的社会监督，自觉把政府权力运行置于阳光之下，有力提高政府工作的透明度。扎实开展党的群众路线教育实践活动、“三严三实”专题教育和“两学一做”学习教育，持续加强政府系统廉政建设、作风建设和党的建设，推动工作作风切实转变，群众满意度调查评价始终保持全省第一。

　　各位代表，过去五年取得的每一项成绩，都是市委坚强领导的结果，是市人大、市政协支持监督的结果，是社会各界齐心协力、全市上下埋头苦干的结果。在此，我代表市政府，向全市人民，向人大代表和政协委员，向各民主党派、工商联和无党派人士、各人民团体，向驻威人民解放军指战员、武警官兵，向各位老领导、老同志，向关心支持威海发展的各界人士和海内外朋友，致以崇高敬意和衷心感谢！

　　各位代表，威海正处在改革攻坚期、发展关键期和社会转型期，前进道路上既有重大机遇和有利条件，也面临不少困难和严峻挑战。经济下行压力依然较大，支持实体经济发展的力度有待加大，方式也需要更加灵活；人才短板仍然是制约发展的瓶颈，科技和金融结合得还不够紧密，创新资源的集聚能力有待提升；产业集群发展的水平不够高，产业链条向“微笑曲线”两端延伸的进程还不够快，新经济、新产业、新技术、新模式发育不足；文化体育产业发展规模不大、结构不优，全域旅游缺少大项目支撑，现代服务业发展层次有待提高；交通、水利等基础设施建设还需加强，城市功能有待进一步完善；部分群众生活还比较困难，社会事业发展还不能完全满足群众需求；安全生产形势严峻，各类风险防范措施有待加强；部分政府工作人员依法办事、高效服务的意识和能力不够强，懒政怠政、推诿扯皮和腐败现象仍有发生。我们一定直面问题，按照“逆向切入”的思路，采取更加有力的措施，切实加以解决。

二、今后五年政府工作的总体目标和主要任务

　　新一届政府履职尽责的五年，是全面建成小康社会的决胜阶段，也是实施“十三五”规划的关键时期。按照市第十五次党代会的战略部署，政府工作的总体要求是：在以习近平同志为核心的党中央领导下，高举中国特色社会主义伟大旗帜，全面贯彻党的十八大和十八届三中、四中、五中、六中全会精神，以邓小平理论、“三个代表”重要思想、科学发展观为指导，深入贯彻习近平总书记系列重要讲话精神和治国理政新理念新思想新战略以及视察山东重要讲话、重要批示精神，统筹推进“五位一体”总体布局和协调推进“四个全面”战略布局，坚持稳中求进工作总基调，牢固树立和贯彻落实新发展理念，适应把握引领经济发展新常态，坚持以提高发展质量和效益为中心，坚持以推进供给侧结构性改革为主线，坚持生态立市、科教兴市，坚持全域城市化、市域一体化和产业强市、工业带动、突破发展服务业，推动各项事业和全面从严治党迈上新台阶，努力提前全面建成小康社会，实现现代化幸福威海建设新跨越。

　　主要目标任务是：提前实现“两个翻番”目标，生产总值、城乡居民收入比2010年翻一番，经济规模和质量效益大幅提高，提前实现全面小康领域、人口、区域全覆盖，城乡文明程度、社会管理水平、公共服务能力、区域生活品质明显提升，加快建设更具活力的创新创业之城、更富魅力的文明幸福之城、更可持续的绿色生态之城，打造更具影响力的宜居、宜业、宜游、宜学的卓越城市。重点实现“六个提高”：

　　（一）提高人民群众幸福指数。坚持以人民为中心的发展思想，把发展硬道理更多体现在增进人民福祉上。实施就业促进和全民参保计划，实现更加充分的就业，让群众享受更高水平的社会保障。深化基础教育综合改革，全面提升学校现代化水平，以提高质量为核心促进各类教育内涵式发展。率先建成全国健康城市，城乡居民主要健康指标全国领先。积极应对人口老龄化，完善社会养老服务体系。深化海陆一体平安建设，打造最具安全感城市。保障群众“吃住行”安全，率先建成国家食品安全示范城市。完善社区文化休闲、体育健身、物业管理等公共服务，打造更加便捷的生活服务圈，不断提高人民群众的满意度和获得感。

　　（二）提高产业发展层次。坚持把改善供给侧结构作为主攻方向，持续激发实体经济企业活力，推动产业迈向中高端水平，实现三次产业协调融合发展。突出先进制造业集群集聚发展，实施千亿产业链培育工程，扶持领军企业和“行业小巨人”，争创“中国制造2025”示范区，加快推动“威海制造”向“威海智造”转变。推动现代服务业加速突破，优化提升全域旅游、商贸流通、现代物流产业，培育壮大现代金融、电子商务、科技信息、医疗健康、文化创意、体育休闲产业。推动国家现代农业示范区建设，突破发展生态农业、休闲农业。深入推进蓝色经济区建设，加快建设国家海洋高技术产业基地，打造海洋经济示范区。

　　（三）提高区域创新能力。加快创建国家创新型城市，推进国家自主创新示范区建设，构建“一区多园”区域创新格局。大力培育具有持续创新力、竞争力的名牌产品和品牌企业，建成全国质量强市示范城市。创建国家军民融合科技协同创新平台，打造军民科技融合示范区。开展“创业威海”行动计划，争创国家“双创”示范基地，构建全链条“双创”生态圈。实施英才计划，聚集更多人才到威海创新创业。积极承接重大改革创新试点，推出一批可复制可推广的“威海经验”。高标准推动各类开发区“二次创业”，实现全方位上档升级，增创开放型、创新型经济发展新优势。

　　（四）提高生态宜居城市品质。按照“中心崛起、两轴支撑、环海发展、一体化布局”的思路，坚持精心规划、精致建设、精细管理，全面提升城市承载功能。加快建设国家产城融合示范区，集中力量建设东部滨海新城，增强各重点区域城市功能。创建生态文明建设示范市，全面加强大气、水、土壤污染治理，环境空气质量稳定达到国家二级标准，让山、海、湾、滩、岛、泉、河焕发生机、更加靓丽。坚持以人的城市化为核心，健全市民化推进机制，推动城乡居民共享更加美好的生活，户籍人口城镇化率达到68%，常住人口城镇化率突破70%。

　　（五）提高基础设施现代化水平。坚持统筹规划、合理布局，有序推进威海机场迁建，积极推进莱西—海阳—乳山—荣成高速铁路工程，推动城市轨道交通规划建设，织密微循环路网，全面提升城乡公交服务能力，持续拓展国际物流大通道，着力构建现代化综合交通运输体系，实现更高水平的外联内畅。实施重点水利工程，构建安全可靠的防洪减灾体系、安全高效的供水保障体系、水清岸绿的流域生态体系。建设城市“十个系统”和农村“七改”工程，提升城乡公用设施水平。实施信息网络设施提升工程、云平台建设工程、信息安全保障工程，推动智慧城市基础设施覆盖城乡，城市管理和服务各领域实现智慧化，让智慧城市建设成果更多更直接惠及人民群众。

　　（六）提高文化软实力和影响力。大力倡导社会主义核心价值观，弘扬中华优秀传统文化，扎实推进文明市民培育工程，培育良好家风家教。推进文明行业、文明社区、文明单位和文明家庭建设，推动荣成市、乳山市建成全国文明城市，巩固全国文明城市建设成果。深入实施“七五”普法规划，培育社会主义法治文化，加强法治城市建设。推进公共文化服务标准化、均等化、数字化，实施历史文化展示工程，讲好威海故事，增强文化自信，传承发扬新时代威海精神，凝聚起奋发有为、干事创业强大动力。

三、2017年政府工作重点

　　各位代表，2017年将召开党的十九大，也是地级威海市建市30周年，做好新一届政府的起步工作意义重大。全市经济社会发展主要预期目标为：生产总值增长8%左右，一般公共预算收入增长9%左右，固定资产投资增长11%左右，社会消费品零售总额增长10.5%左右，进出口保持稳定增长，城镇和农村居民人均可支配收入分别增长8%和8.5%左右，居民消费价格涨幅控制在3%左右，城镇登记失业率控制在2%以内，完成省下达的节能减排约束性指标。重点做好以下工作：

　　（一）全力振兴实体经济。树立实体为本理念，突出制造业转型升级，与企业一道攻坚克难，提升实体经济发展活力和水平。

　　持续降低实体经济企业成本。全面落实小微企业享受减半征收所得税范围扩大、科技型中小企业研发费用加计扣除比例提高等结构性减税政策，支持企业用好政策红利。进一步降低企业非税负担，严禁搭车收费、变相收费、强制收费，最大限度为企业减负让利。做好生育保险与职工基本医疗保险合并试点工作，降低企业社保缴费负担。创建冷链物流示范城市，提升物流服务保障能力，降低企业流通成本。完善金融机构支持实体经济绩效评价奖励政策，鼓励金融机构创新金融产品，更好支持实体经济发展。继续发挥科技支行、服务贸易支行和小微企业贷款风险补偿基金作用，破解企业融资难题，促进更多金融活水流向实体经济。

　　实施创新驱动发展战略。落实企业研发投入财政补助、科技创新券补助等政策，完善促进科技和金融结合政策，引导企业建立研发准备金制度，带动提高全社会研发投入总量和强度。更好发挥工信部电子信息技术综合研究中心等重大创新平台作用，鼓励行业骨干企业牵头成立技术创新联盟5个以上，支持企业建设研发机构，开展产学研协同创新。实施新型研发机构培育计划，支持高校建设科技园、创业园。深化小微企业“一企一技术”，培育提升行业单项冠军。开展知识产权预警导航，培育省级知识产权示范企业20家以上。鼓励发展科技中介服务机构，支持企业开展技术交易，加快科技成果引进和转化。制定人才发展体制机制改革意见，举办国际英才创业峰会，集聚“高精尖”人才。

　　实施质量强市和品牌战略。开展增品种、提品质、创品牌“三品”专项行动，推进内外销产品“同线同标同质”，扩大高质量产品和服务供给。大力实施标准化战略，支持企业主导或参与制定国家和国际标准。引导企业有效运用商标国际注册，加快品牌国际化进程。推广全面质量管理，推动企业设置首席质量官，夯实企业质量管理基础。强化品牌培育和保护，促进老品牌叫得更响、大品牌走得更远、新品牌在海内外闯出更广阔的天地。

　　深化供给侧结构性改革。坚持用改革的办法，深入推进“三去一降一补”。加快淘汰落后和过剩产能，引导造船、轮胎、玻璃行业重点企业提高产能利用率。引导优势企业、优质资本兼并重组“僵尸企业”。推动化工企业进园区。科学调控土地供给，分类施策推进房地产去库存，缩短房地产去化周期。推进公租房和棚户区改造货币化安置，争取货币化安置率超过50%。加强住房市场监管和整顿，规范开发、销售、中介等行为，促进房地产市场平稳健康发展。

　　助推各类企业梯次发展。落实“个转企、小升规、规改股、普升高”扶持政策，支持骨干企业快速膨胀，成长型企业加速发展。实施高新技术企业培育成长计划和“千帆计划”，年内新增科技型企业400家。实施服务业优势企业培育工程，支持200家具有行业和区域代表性的服务业企业做强做精。推进人力资源服务产业园建设，扩大创业大学培训规模，提高培训质量。加强创新创业孵化企业跟踪服务，新增科技孵化企业150家、创业孵化企业610家以上，支持大众创业、万众创新，让更多实体经济的种子生根发芽、开花结果。

　　各位代表，振兴实体经济，需要一大批崇尚创新、追求卓越的优秀企业家，需要一大批爱岗敬业、精益求精的“威海工匠”。企业家是宝贵的社会财富，要尊重企业家，关心企业家，稳定企业家信心，支持企业家专心创新创业。做好企业需要匠人，做优产品呼唤匠心，精益求精的工匠和企业家一样应当受到尊重。要大力弘扬企业家精神和工匠精神，培育和壮大实体经济发展的中坚力量，推动威海经济向质量型、品牌型阔步迈进。

　　（二）推进产业转型升级。坚持“产业强市、工业带动、突破发展服务业”，加快培育战略性新兴产业和高端成长型产业，进一步优化产业结构。

　　加快推动制造业改造升级。出台推进工业经济转型升级的意见。降低购置设备贴息门槛，提高补助比例，鼓励企业实施高水平技术改造。实施118项重点工业投资项目，扩大有效投资。突出专业特色，扎实推进五大重点创新产业园建设，引导骨干企业、重点项目和创新资源向园区集中，提升优势产业协作配套能力，促进战略性新兴产业集聚发展。开展智能制造专项行动，重点突破工业机器人、高档数控机床、智能测控装置等智能制造产业。

　　积极推动现代服务业突破发展。坚持“敲开核桃、一业一策”，推动服务业模式创新，培育壮大服务业特色产业。大力发展健康产业，规划建设国际医疗健康产业城，推动医疗服务、健康养生、文化体育等多业态融合发展。加快建设软件和服务外包产业园，支持发展信息技术服务产业。实施休闲经济培育计划，创建国家全域旅游示范区，推动乡村旅游提档升级，突破发展工业旅游，形成多元休闲产品供给结构。开发户外运动项目，举办铁人三项世界杯赛，做活做精帆船、垂钓等品牌赛事，打造最佳户外休闲运动城市。

　　扎实推动农业供给侧结构性改革。狠抓农产品标准化生产、品牌创建、质量安全监管，加强农产品质量安全可追溯体系和科技云平台建设，增加绿色优质农产品供给。实施农药化肥零增长行动，发展节本农业和水肥一体化。大力发展现代果业、特色养殖业，拉长特色农业发展链条。深化供销社综合改革。积极培育新型经营主体，培养新型职业农民，拓展农民增收致富空间。

　　统筹推动海洋经济转型发展。加快海洋牧场建设，培育海洋食品品牌，让威海刺参、乳山牡蛎、荣成海带等地理标志品牌叫得更响。突破休闲渔业发展，打造综合性休闲渔业基地和海上田园观光体验区20处以上。推行以港管船制度，多措并举促进渔民转产转业。推进海洋生物遗传育种中心、远洋渔业综合基地建设。发展海洋高端生物制品产业，促进钓具产业转型升级，推动海洋装备制造业高端化发展。

　　各位代表，产业跨界融合是大势所趋，要因势而谋、顺势而为，主动发现和培育跨界融合形成的新产业新业态，努力拓展产业发展新空间。推动农村一二三产业加速融合，开展农村产业融合发展试点，积极培育“新六产”。推动制造业与服务业深度融合，鼓励企业建设工业设计中心和品牌运营中心，支持企业推进个性化定制，培育壮大服务型制造。实施“互联网+”行动计划，大力发展农村电商，引导企业扩大电子商务应用，促进传统产业拥抱互联网，插上腾飞的翅膀。

　　（三）全面深化改革开放。扎实推进重点领域改革，不断增强市场活力、社会创造力和开放竞争力。

　　推动对外开放先行先试。积极融入“一带一路”战略，统筹推进中韩自贸区地方经济合作示范区、国家服务贸易创新发展试点工作。加快推进综合保税区配套基础设施和监管设施建设，加强国际贸易“单一窗口”建设和推广应用，提高贸易便利化水平。培育外贸新业态新模式，提升外贸综合服务平台服务能力。实施百展市场计划，大力拓展新兴市场，构建多元化市场格局。扩大先进技术、关键设备进口。坚持招商引资与招才引智紧密结合，增强精准招商实效。开展点对点招商，着力引进世界500强企业和行业领军企业。支持企业并购重组，加强国际产能合作。

　　深化企业改革。进一步规范国有企业管理。推进机关事业单位所属企业脱钩和分类管理。开展混合所有制改革试点。加强各类国有资本投资运营公司管理，推动政府引导基金加快投资运作。推进规模企业规范化公司制改制，加快建立现代企业制度，新增改制企业260家以上。依法保障各种所有制经济组织和公民财产权，激发各类市场主体创新创业积极性。

　　推动财税领域改革。深化预算绩效管理改革，提高财政资金使用效率。理顺市县两级政府财政事权和支出责任配置。深化国库集中支付改革，全面推行电子化管理。健全公共财政体系，严格控制“三公”经费等一般性支出。分领域、分阶段缩小县域人均基本公共服务支出水平差距，逐步实现“五个基本统一”。推广政府与社会资本合作模式，激发民间投资活力和潜能。

　　推进金融改革创新。加快对接多层次资本市场，按照“改制一批、培育一批、申报一批、上市一批”的思路，推动更多企业到资本市场上市，推动中小企业在新三板和区域性股权交易市场挂牌。培育挖掘债券发行主体，推动更多企业发行公司债、企业债和各类债务融资工具。支持有实力的私募基金管理机构来我市设立股权投资基金。大力发展普惠金融，引导金融机构加大对“三农”和小微企业的支持力度。支持融资性担保机构发展，提高小微企业增信能力。扩大小额贷款保证保险覆盖面，探索开展科技保险。推动蓝海银行开业运营。促进小额贷款公司、民间融资机构规范发展。开展互联网金融风险专项整治清理行动，利用新媒体加强涉众型经济案件预警宣传，强化高利贷风险防范，守住不发生系统性、区域性金融风险底线。

　　扎实开展国家新型城镇化综合改革试点。出台人口市民化规划，年内就地转移农业人口3万人以上，进一步提高城镇化率。深化农村产权制度改革，落实“三权分置”促进适度规模经营，稳妥推进农村集体经营性资产股份合作制改革。扎实开展大中小城市和新生小城市试点，抓好特色小镇和重点示范镇建设。

　　（四）推进生态宜居城市建设。坚持建管并重，不断完善城市功能，提升城市承载力和宜居度。

　　加强生态环境保护。实施能效“领跑者”制度和工业绿动力计划，促进工业领域煤炭清洁高效利用。加强散煤综合治理，加快淘汰分散燃煤锅炉，全面完成燃煤热电机组超低排放改造任务。开展加油站清理整顿和油品质量专项检查，强化机动车尾气、餐饮业油烟、扬尘污染防治。全面落实河长制，加强重点河流综合整治。实施工业点源深度治理、农业面源污染防治。加强集中式饮用水水源地环境保护，整治畜禽养殖场67处。加快雨污分流设施建设。推进市属热电企业中水利用工程。大力倡导节水理念，推广高效节水技术，推动生产生活节水护水。严格落实海岸带执法巡查制度，开展蓝色海湾整治行动。开展入海排污口调查，实施水质自动在线监测。严格依法用地管地，加快清理处置闲置和低效利用土地。开展土壤污染调查与监测，健全固体废物处置设施。加快推进生活垃圾分类收集。加强餐厨废弃物统一处理。整治噪声污染。加大森林资源、湿地资源保护力度，加强自然保护区、风景名胜区、海洋保护区等生态功能区建设。开展自然资源资产离任审计，促进生态文明建设责任落实。

　　统筹城乡基础设施建设。加快建设环山快速路、文莱高速公路、乳山口大桥等重点项目。推进龙山湖等24项重点水利工程建设。实施48项城建重点工程。统筹城市地上地下建设，实施好地下综合管廊项目，加快建设海绵城市。推进美丽乡村标准化建设和示范创建活动，新建农村社区服务中心165个，开展普惠式农村供暖试点，实施农村无害化卫生厕所改造8.6万户，改造农村公路150公里，改善418个村、22.5万名群众饮水条件，重点解决283个定时供水、临时停水的缺水村供水保障问题。

　　提升城市管理水平。加强城市规划管理，编制城市风貌保护规划。推动城市管理标准化建设。理顺城管执法体制机制。扎实开展城市违法建设治理行动，持续推进铁路沿线环境综合整治。完成38个老旧住宅小区综合整治，改造棚户区1.9万户。推进18个智慧示范项目，促进民生服务智慧应用线上线下集成融合。推进建筑业改革发展，提高设计水平和工程质量。办好第九届中国威海国际人居节及国际建筑设计大奖赛。

　　（五）织密扎牢民生保障网。坚持民生优先，增加优质公共服务供给，办好28件民生实事，解决好群众关心的实际问题。

　　巩固脱贫攻坚成果。实施贫困村整体提升工程，加强扶贫产业项目建设，推动金融、教育、健康扶贫，争取年内省定扶贫工作重点村全部达到退出标准。实施最严格的评估考核，防止假脱贫、“被脱贫”、数字脱贫。建立基本生活保障协调机制，加强困难群体临时救助，实施重特大疾病医疗救助，进一步提高城乡低保标准，切实做好托底工作。

　　加强就业和社会保障。落实就业援助制度，加强对困难人员的就业援助，统筹推进高校毕业生、农村劳动力等各类群体就业，新增城镇就业3.5万人。加强劳动保障监察和争议调解仲裁，切实解决好农民工工资拖欠问题。推动养老保险制度改革，完善居民大病保险政策，建立职工大病保险、职工长期护理保险制度。提高居民医保补助标准，探索开展基本药物全额保障试点，推进跨省异地安置退休人员住院医疗费即时结算。实施政府救助保险，完善灾害保障救助体系。

　　提升教育发展水平。全面推进基础教育、学前教育省级改革试验区建设。新建改扩建学校18所，新增学位2万个，基本解决城镇中小学“大班额”问题。新建改扩建普惠性幼儿园15所，新增学位3600个。加强中小学体卫艺教育，促进家庭教育和学校教育相结合。推进中小学德育课程一体化，加强中华传统文化教育。开展中小学“小班化”教学试点。全面开展城区小学生课后免费托管工作。积极适应新高考改革，推动普通高中“选课走班”教学。实施高等职业教育创新发展行动计划，推进中等职业学校品牌专业与实训基地建设，增加现代学徒制试点。实施特殊教育提升计划。加快推进威海一中新校区、市青少年活动中心、威海艺术学校等新建改造工程，完成市职业中等专业学校整合搬迁，启动建设市中小学生综合实践教育中心三期工程。

　　加快健康城市建设。深化公立医院综合改革，推进分级诊疗制度建设，建立家庭医生签约服务机制。加快国家中医药综合改革试验区建设，加强中医药传统文化传承教育，新增国医堂25处。开展“三减三健”行动，有效提高慢性病防控水平。完善计划生育服务管理，健全孕妇产前筛查链条。开展国家级医养结合试点，推广政府购买社区居家养老服务模式。新建社区养老服务中心10处、农村幸福院100处，新增各类机构养老床位3000张。开展残疾人精准康复服务行动。完善公共体育服务体系，建设市游泳馆，新建城市健身步道50公里、镇级全民健身中心24处。办好市第五届运动会。

　　扎实推进文化建设和文明创建。大力发展文化产业园区、骨干文化企业和文化产业项目，培育新型文化业态，推动文化与相关产业融合发展。加快建设市图书馆新馆、群众艺术馆新馆，推动基层综合文化服务中心覆盖所有村居。试点建设“城市书房”，实施全民阅读推广计划。推进城乡儒学讲堂建设，加强优秀传统文化弘扬传承和教育普及。推动历史文化镇村保护开发，支持建设镇村历史文化展馆。加强胶东威海党性教育基地建设。推进县级综合档案馆建设。加强文化市场监管。组织好建市30周年系列活动。开展“爱文明家园、做文明市民”三项行动，深化乡村文明行动，倡树文明社会风尚，进一步提高文明城市建设水平。

　　加强和创新社会治理。扎实推进全国社会信用体系建设示范城市创建工作。创新矛盾纠纷排查调处机制，推行人民调解网格化管理。支持人民法院解决执行难问题。推动警务助理、法律顾问进村进社区。深入实施“天网工程”。严厉打击非法传销。健全安全生产风险分级管控与隐患排查治理体系，强化森林防火、渔船和渔业安全管控，坚决防范遏制各类重特大事故发生。深化“平安行·你我他”行动。开展餐饮业质量安全提升工程，实施食品小作坊、小餐饮、食品摊点“三小”规范提升行动。完善风险防控与应急处置体系，增强城市抗风险能力。开展“阳光慈善”行动。强化双拥共建和优抚安置，加强国防动员、民兵预备役、打击走私和人民防空建设。依法保障妇女、儿童、老年人、残疾人合法权益。做好民族、宗教、外事、侨务、对台、仲裁、史志、地震、气象、对口支援和扶贫协作工作。

四、政府自身建设主要任务

　　牢固树立政治意识、大局意识、核心意识、看齐意识，坚决维护以习近平同志为核心的党中央权威，把全面从严治党要求落实到从严治政之中，大力建设法治政府、服务型政府、廉洁政府。

　　（一）深入推进依法行政。深入贯彻全面依法治国要求，把法治理念贯穿政府工作全过程。深入推进简政放权、放管结合、优化服务改革，加快转变政府职能。动态调整“四张清单”，向基层下放真正管用的权力，把该“接”的接到位，把该“放”的放彻底，把该“清”的清干净，以行政权力瘦身为市场主体强身。强化整体政府理念，推进机构整合，优化行政资源配置。加强城乡建设与管理、环境保护等重点领域政府立法工作。落实重大行政决策“五项制度”，发挥政府法律顾问作用，保证政府决策依法合规。加大行政复议纠错力度，及时化解行政争议。

　　（二）积极转变监管方式。坚持“宽进严管”，构建与简政放权要求相适应的常态化事中事后监管机制，提高政府管理的“含金量”和“精准度”。建设网格化监管平台，推进跨部门跨行业综合执法，清理职责交叉和分散事项。全面推行“双随机、一公开”监管模式，加快构建大数据监管模型，进一步提高行政执法效能。健全行政执法监督机制，严格公正文明规范执法，有效遏制权力寻租和执法腐败。

　　（三）持续优化政务服务。严格落实行政机关首问负责制，健全部门协作机制，协调联动办理企业和群众诉求。倡树“为企业节约时间就是为企业节约成本”的理念，实施政府提速工程，进一步提高行政服务效能。加快推动“互联网+政务服务”，推进“一窗式”“一门式”服务模式改革，建设全市统一的电子证照库，将电子签章、网上支付等引入云审批，实现零触式服务。扎实开展联系服务企业行动，走上门去送政策、摸情况、听意见、解难题。改进和创新联系群众机制，推动公共服务向镇村和社区延伸，打通政策落地的“最后一米”，让幸福在群众家门口升级。

　　（四）切实加强政风建设。坚持干字当头、真抓实干，进一步健全督查问责机制，以“钉钉子”精神抓好各项工作落实。反对形式主义，力戒以会议贯彻会议、以文件落实文件，杜绝形象工程，反对统计数据造假。全面推行政府权责清单、小微权力清单、负面言行清单，从源头上防范不作为、慢作为、乱作为，整肃懒政怠政。办理民生实事尽力而为、量力而行，不浮夸、不忽悠、不作秀，以实实在在的工作成效造福于民、取信于民。

　　（五）自觉接受各方监督。坚持内部监督和外部监督相结合，健全依法接受监督工作机制。落实政府重大决策出台前向本级人大报告制度，积极主动向人大及其常委会报告工作，强化政协民主协商，认真办理人大代表建议和政协提案，主动在社会和舆论监督下开展工作。强化行政监察和审计监督，加强公共资源交易、政府投融资、公共基金、国有资本等重点领域监管，健全覆盖各领域、全过程的内控机制。

　　（六）严格遵守廉洁纪律。坚持把纪律和规矩挺在前面，敬畏权力，严守底线，大力推进“廉洁威海”建设，营造崇廉尚洁、向上向善的浓厚氛围。严格落实全面从严治党责任，从严从实抓好巡视“回头看”整改。坚持惩防并举、标本兼治，加强党风廉政教育和岗位廉政风险防控，健全纠治“四风”长效机制，防范群众身边的“微腐败”，无禁区、全覆盖、零容忍惩治腐败行为。

　　各位代表，建设法治政府、服务型政府、廉洁政府，人民满意是最根本的目标。我们一定始终牢记责任担当，以对党和人民绝对忠诚的态度，实打实地兑现各项承诺；始终紧贴群众脉搏，把群众呼声作为第一信号，主动走到群众中间，“零距离”交流，充分了解社情民意，与群众换位思考，使政府工作更加接地气；始终严守底线红线，从严从实加强政府系统干部队伍管理，以刚性的纪律、过硬的作风，赢得群众的信任和支持；始终自觉摆正位置，以人民为尊、以人民为重，把政府工作的监督权、评判权交给群众，向党和人民交出一份合格答卷！

　　各位代表，带着人民群众的梦想和期待，我们踏上新的征程。让我们更加紧密地团结在以习近平同志为核心的党中央周围，在省委、省政府和市委的坚强领导下，把责任与使命扛在肩上，不忘初心、继续前进，干在实处、走在前列，为提前全面建成小康社会、实现现代化幸福威海建设新跨越而努力奋斗，以实际行动迎接党的十九大胜利召开！
